# Case report: Remarkable efficacy of negative-pressure wound therapy in giant lower extremity elephantiasis neuromatosa for vascularization, skin grafting, and fluid control

**DOI:** 10.1016/j.ijscr.2024.109428

**Published:** 2024-02-22

**Authors:** Lisa Y. Hasibuan, Almahitta Cintami Putri, Graciella Novian Triana Wahjoe Pramono

**Affiliations:** Division of Plastic Reconstructive and Aesthetic Surgery, Department of Surgery, Faculty of Medicine, Universitas Padjadjaran, Bandung, Indonesia

**Keywords:** Neurofibromatosis, Infected neurofibroma, Negative pressure wound therapy, Plexiform neurofibroma, Elephantiasis neuromatosa

## Abstract

**Introduction and importance:**

Neurofibromatosis type 1 is a benign peripheral nerve tumor, often manifests as plexiform neurofibroma that may cause severe dysfunction, pain, and disfigurement. Bleeding has been reported as a complication of plexiform neurofibroma due to vascular fragility and vasculopathy that may develop into life-threatening bleeding especially after excision procedure. Consequently, post excision complications also include dehiscence and infection.

**Case presentation:**

We report a 23-year-old male with elephantiasis of the left lower extremity due to giant plexiform neurofibroma who underwent preoperative embolization followed by serial surgical mass reduction. There were postoperative complications consisting of hematoma, wound dehiscence, and infection.

**Clinical discussion:**

Negative pressure wound therapy is often used to accelerate wound healing, including infected wounds. However, negative pressure wound therapy has been a debatable modality for wound care of neurofibroma due to reported risks of profuse bleeding during its use.

**Conclusion:**

In this case, despite the size, negative-pressure wound therapy has shown good results for infected neurofibroma wounds and as an adjunct as wound dressing for defect closure of neurofibroma with split-thickness skin graft.

## Highlights

• Neurofibroma is a tumor with vasculopathy and lymphatic abnormalities
• Negative Pressure Wound Therapy is commonly used for complicated wounds, but publication of use in highly vascularized wounds is limited
• Use of Negative Pressure Wound Therapy with an elephantiasis neuromatosa patient with extremely large wound, bleeding and exudates proved to be successful

## 1 Introduction

Neurofibromatosis type 1 or Von Recklinhausen Disease is a multisystem, autosomal dominant disorder. About 30–50 % of neurofibromatosis type 1 patients have plexiform neurofibromatosis, a peripheral nerve sheath tumor that often causes severe disfigurement and dysfunction [1,2]. Postoperative hematoma and bleeding are common in plexiform neurofibromas, and can lead to ulcer formation and other complications. Negative pressure wound therapy or commercially known as V.A.C. are a debatable wound care modality in these cases because some publications report an increased risk of bleeding, and some reports of it being used as hemostatic adjuncts [3,4]. In this study we present a 23-year-old male with neurofibromatosis type 1 who underwent preoperative embolization followed by serial surgical mass reduction. There were postoperative complications consisting of hematoma, wound dehiscence, and infection. The patient underwent subsequent negative pressure wound therapy (NPWT) and split-thickness skin grafts with successful results. In this case study, we can observe that negative pressure wound therapy with the proper setting and conditions would be a hemostatic adjunct as well as an exceptional modality for infection control.

## 2 Case presentation

A 23-year-old male presented to our Plastic Surgery outpatient clinic with a grotesque overgrowth of soft tissue on his left lower extremity that had begun to grow during his teenage years (around 16 years old). None of his known relatives have any similar conditions.

**Fig. 1:**
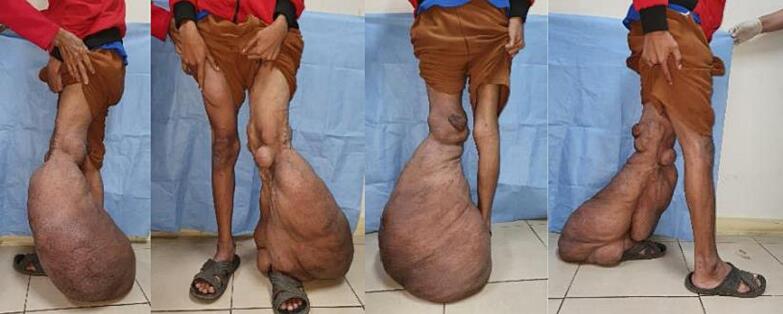
Clinical appearance of elephantiasis due to giant overhanging plexiform neurofibroma of the patient's left lower extremity. Patient was able to walk, although burdened by the large mass.

**Fig. 2:**
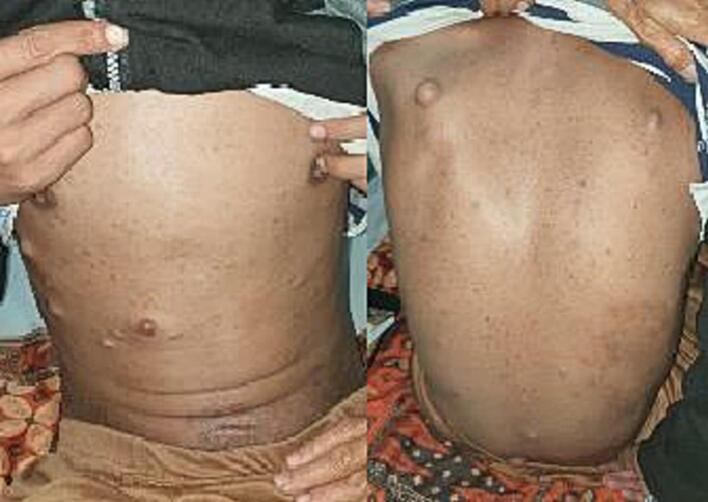
Multiple neurofibromas and café-au-lait macules located on the anterior and posterior trunk. These signs and symptoms fulfil the criteria required for this patient to be diagnosed Neurofibromatosis type 1.

Physical examination showed a bulging soft mass spanning the distal third of his thigh up to his left ankle (Fig. 1). The size of the mass was 90 cm × 50 cm × 10 cm. Gigantism of the right lower extremity and swelling mainly at the heel and dorsum of the foot. There were lymph node enlargements of the ipsilateral inguinal lymph nodes. The patient had trouble walking due to the size and weight of the overhanging mass, but he was still mobile. He also had café-au-lait macules and smaller solitary cutaneous and subcutaneous neurofibroma lesions on his anterior and posterior trunk (Fig. 2). Routine laboratory tests were normal. The patient did not have any history related to the central nervous system such as cognitive impairment, epilepsy, or attention deficit hyperactivity disorder.

The patient underwent his first planned surgery for serial mass excision. Prior to the surgery, we had performed embolization of a branch of the feeding artery from the popliteal artery and one from the anterior tibial artery. We then excised several sections of the plexiform neurofibroma to reduce the mass and to acquire a sample for biopsy. Histopathological examination showed overgrowth of peripheral nerve components and connective tissue consistent to neurofibromatosis. His first surgery was uneventful, and the patient was discharged 5 days after the surgery.

MRI examination of the patient's extremity showed a soft tissue mass on his left leg which infiltrate surrounding muscles. It also showed high vascularization and large calibre blood vessels on the mass and the proximity of the main arteries of the leg.

**Fig. 3:**
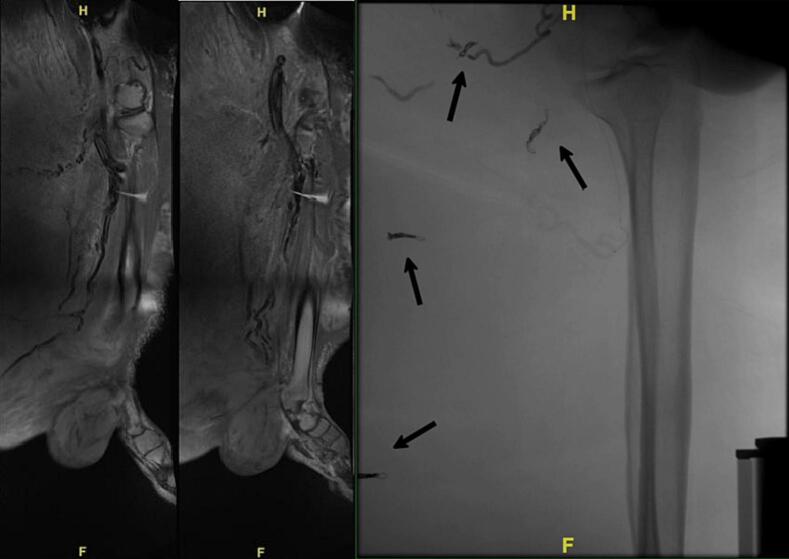
(A) MRI of the patient's left lower extremity, showing a mass infiltrating the gastrocnemius, soleus, peroneus longus, and brevis muscles. Hypervascularization was shown surrounding the mass in close proximity to important vessels of the lower extremity. (B) Embolization of the feeding branches of the popliteal and peroneal arteries. A total of four branches were embolized, shown by the straight arrows.

The patient was re-admitted for the next stage of excision surgery six months after, again with pre-operative embolization. Embolization was done on the feeding branches of the popliteal and peroneal arteries (Fig. 3). Proximal mechanical embolization with coiling technique was used.

**Fig. 4:**
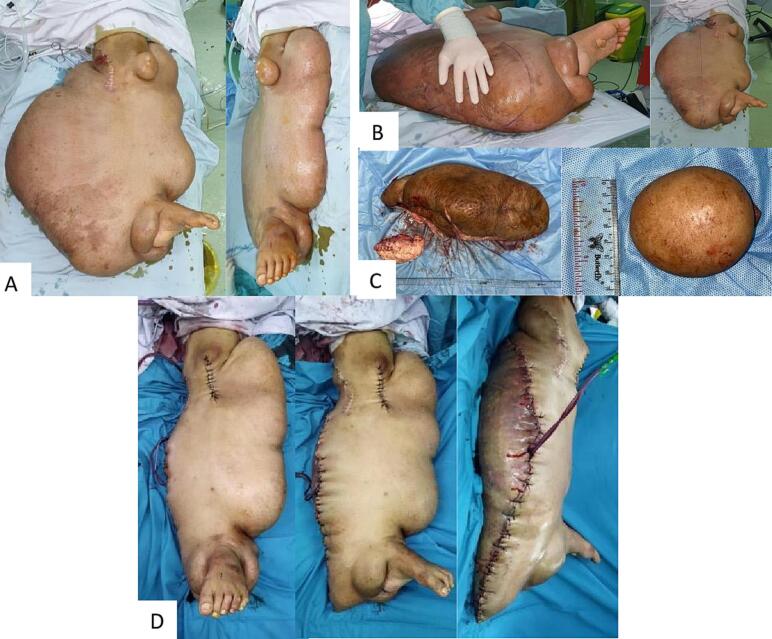
(A) Preoperative picture of giant neurofibroma of the left lower extremity (B) Lateral wedge excision design of the large mass and excision of solitary neurofibroma near the left knee (C) Excised mass (the larger mass size was 90 × 25 × 10 cm, and the smaller solitary mass was 6 × 5 × 3 cm) (D) Postoperative picture of the patient after primary suture and placement of vacuum drain.

On the next surgery, the patient underwent a large hemi-resection of the mass, size of the excised mass was 90 × 25 × 10 cm (Fig. 4). A wide wedge-shaped excision was performed for the mass, considering neurofibromatosis is a very fragile lesion with a possibility of skin necrosis. Through this wide wedge shape, we hoped to excise as much mass as possible while making sure that skin vascularization is maintained by conserving sufficient subcutaneous tissue. Intraoperative haemostasis was performed using cauterization, ligation and absorbable hemostatic agents (Spongostan®). Then the wound was closed primarily with silk 2-0 (Silkam®), and a vacuum drain was placed. The postoperative wound was dressed with conventional impregnated gauze and elastic bandage.

Before the procedure, the patient underwent the process of obtaining informed consent regarding the impending intervention. This step is crucial, especially because of the risks of the extremely large mass excision leading to large amounts of blood loss and increased risk of complications. Additionally, the patient was also provided with informed consent regarding potential publication and this work has been reported in line with the SCARE criteria [5].

**Fig. 5:**
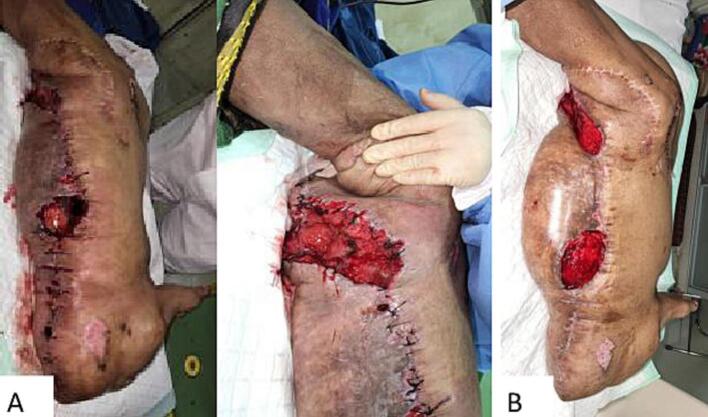
(A) Picture showing wound dehiscence, necrotic tissue, hematoma formation, and wound infection during conventional wound care. (B) Wound 5 days after surgical debridement, intravenous antibiotics and wound care using NPWT showed healthy granulation tissue, no hematoma, and resolved infection.

For five days post op, the patient produced large amounts of blood in the drain, reaching up to 500 cc of bloody discharge per day. There were no signs of active bleeding from a solitary blood vessel, but lymphatic exudate was observed. Wound care in the ward showed hematoma formation and wound dehiscence with foul purulent pus production (Fig. 5). Pus culture resulted with a growth of *Escherichia coli*, susceptible towards Gentamycin, Amikacin, and Tigecycline. The patient was given intravenous antibiotics. The hematoma and fluid accumulation with wound dehiscence was suspected due to dead space in the post-excision wound caused by inadequate soft-tissue contact. The patient was taken back to the operating theatre, where we explored and debrided the wound. Intraoperatively we found necrotic tissue with pus, heavy exudate and blood clots. Subsequent wound care was performed with continuous negative-pressure wound therapy with a low pressure of 75 mmHg.

**Fig. 6:**
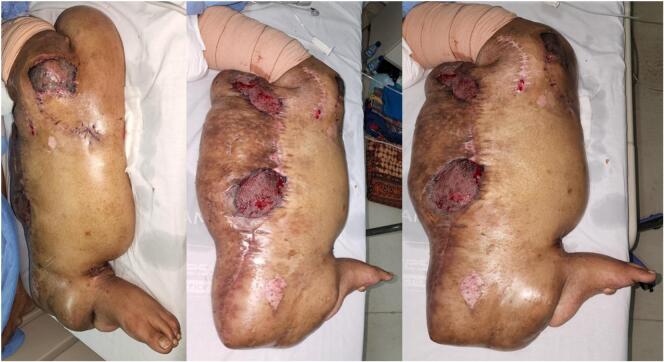
Clinical findings 5 days after re-debridement, split-thickness skin graft, and NPWT showing good graft take and minimum raw surface. Patient was then discharged with conventional wound care.

During wound care with NPWT, healthy granulation tissue finally started to grow, infection had subsided, and there were no longer massive amounts of drainage (Fig. 5). After one week, split-thickness skin grafting was performed. Once more, the wound was treated with continuous negative-pressure wound therapy with a low pressure of 75 mmHg. The patient was discharged four weeks after excisional surgery and conventional wound care was continued at home (Fig. 6).

**Fig. 7:**
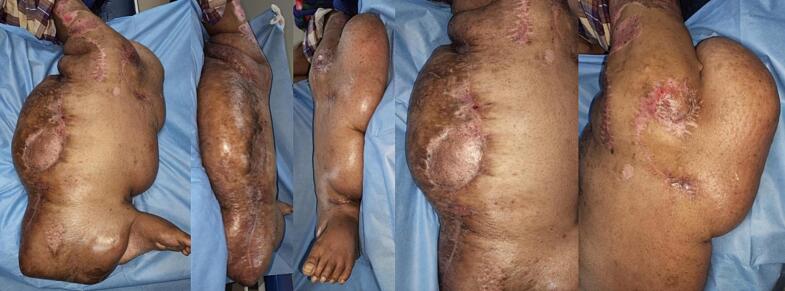
Clinical picture of left lower extremity four months post-surgery.

Our next plan for the patient was to reduce more of the mass as close as possible to the normal structures to obtain a more natural size and better functional movement. However, the patient was lost to follow up possibly due to personal satisfaction with mass reduction and monetary limitations to be able to travel back to our hospital. Four months post operatively, function of the leg was normal, similar to preoperative conditions (Fig. 7). The patient was able to walk but still burdened by the size of the tumor although there was significant weight reduction.

## 3 Discussion

Neurofibromatosis type 1 is a multisystem, autosomal dominant disorder where 30–50 % of the patients have plexiform neurofibroma, a peripheral nerve sheath tumor that may cause severe disfigurement and dysfunction [1,2]. Common differential diagnosis of plexiform neurofibroma includes sarcoma, lymphatic malformation, and venous malformation. Diagnosis of NF1 is usually clinical, where the patient meets a minimum two out of the seven diagnostic criteria developed by the National Institute of Health (NIH) [6]. We observed two clinical criteria in this patient: plexiform neurofibroma and café-au-lait macules and has therefore diagnosed this patient with neurofibromatosis type 1. The giant plexiform neurofibroma in this patient caused an elephantiasis deformity of the left lower extremity and significantly hindered the patient's movement.

Management of plexiform neurofibroma is mainly surgical, which aims to reduce the mass, reconstruct the deformity, and avoid malignant transformation. Furthermore, NF1 is associated with vascular fragility and vasculopathy, thus carrying an increased risk of haemorrhage [7]. Hence, surgical resection of plexiform neurofibroma would require careful preoperative preparation, meticulous intraoperative haemostasis, and close postoperative observation. Preoperative embolization has been reported to be effective in controlling bleeding [7]. This patient had previously undergone surgery with preoperative embolization for diagnostic resection of the mass, followed by an uneventful postoperative care. Preoperative embolization was also done before the second surgery however after the second surgery skin necrosis and infection was observed.

Due to the size of this giant plexiform neurofibroma, haemostasis during surgical resection was a preoperative concern. Hemorrhages may be caused by friable blood vessels due to arterial dysplasia or vascular invasion in to the neurofibroma [8]. The diagnostic angiography confirmed multiple vessels supplying the tumor and selective proximal embolization was done. To avoid skin necrosis associated with embolization, proximal mechanical embolization with coiling technique was used. Similar to a report by Jones et al. and Vélez et al., coils were placed proximally in a major feeding vessel [8,9]. There is no evidence regarding size indication for embolization before plexiform neurofibroma surgery. However due to the intense vascularization and capillary fragility, plexiform neurofibroma would benefit by preoperative embolization unless there are contraindications to the procedure [10].

In this case lymphoscintigraphy was not done. However in a previous study it was found that in an elephantiasis neurofibroma, lymphatic alterations and consequent lymphedema was observed [11]. Lymphoscintigraphy showed dermal backflow in the affected limb, hypertrophy of ipsilateral nodes and a lymph flow delay. With a congenital alteration in the lymphatic network, there would be an increase in lymphatic fluid exudates in the wound, further complicating the wound healing process.

However, in the second surgery there was postoperative hematoma formation with large amounts of blood drainage daily, as well as consecutive wound dehiscence and infection. This was possibly due to the significantly larger size of the resection. We used conventional impregnated gauze and elastic bandage for initial postoperative dressing, which was apparently inadequate to manage a wound of this calibre. Negative-pressure wound therapy is commonly indicated for complex non-healing wounds such as dehisced wounds, secondary ulceration, as well as infected wounds [11]. NPWT is effective towards complex wound by promoting the formation of granulation tissue within the wound, removing excess fluid, promoting blood perfusion and contracting the edges [12]. In a publication by Jones DA et al., they have observed the benefits of NPWT in all 20 patients with infected wounds. The localized use of NPWT for infected wounds includes wound drainage, angiogenesis stimulation, proteinase excretion and decreased local and systemic bacterial load [13]. In this patient we observed benefits from NPWT including controlling exudate and hematoma, decreasing the size of the wound, angiogenesis stimulation and helping the skin graft stick to the wound bed. Application of NPWT on the lower extremity is common and relatively simple in our institution, however the difficulty in this patient is due to the size and weight of the extremity.

Several publications have reported effective usage of NPWT for neurofibromatosis ulcer management, but there were still concerns regarding exacerbated bleeding caused by negative pressure [1,4]. Due to a lack of consensus or guideline, we conducted intraoperative debridement and exploration of the wound. We found no sign of exposed vasculature or active bleeding from isolated blood vessels. We applied NPWT at a relatively low pressure of −75 mmHg, the same pressure previously reported as being effective for two cases of neurofibromatosis [4]. In our case, continuous pressure at −75 mmHg showed no promotion of bleeding, sufficiently contracted the wound, and the patient felt no pain. Kheirabadi B. et al. stated that NPWT can be a hemostatic adjunct with standard hemostatic dressing to control coagulopathic haemostasis by applying pressure to occlude and constrict small blood vessels [3]. This finding was done on porcine models with large, actively bleeding wounds, coagulopathy and hypothermia, where NPWT was used with very high pressure for haemostasis (approximately −500 mmHg). Average haemostasis was achieved in 25–34 min, with less blood loss and fluid resuscitation requirement. Sekiyama et al. used negative pressure for a small-sized neurofibroma ulcer, and had successful results [4]. However, there were only few reports of NPWT used on larger wounds, and not as large as the wound observed in this case [1,12].

We applied −75 mmHg, low pressure VAC in our case as a strategy to avoid excessive bleeding and to be cautious, while observing any signs of bleeding. In a report by Ren et al., bleeding was observed in a patient with −150 to −400 mmHg setting and due to high suction energy, an excessive 1.5 l of fresh blood was immediately found in the drainage bottle [14]. Although a consensus isn't available for NPWT use in bleeding patients, vacuum therapy is contraindicated for exposed arteries. We would still recommend the use of NPWT for small fragile subcutaneous blood vessels.

The wound bed showed healthy growth of granulation tissue, resolution of bleeding and infection, and wound edge constriction after surgical debridement and NPWT. Afterwards, the wound was able to support graft where NPWT was used again as postoperative wound dressing. In this case, NPWT has shown to be effective for infected neurofibroma wounds and as an adjunct as wound dressing for defect closure of neurofibroma with skin grafts. There is still no guideline available on the use of NPWT for post-excision neurofibroma: the optimum duration, pressure, and suction mode. Further studies on neurofibroma post-excision management with NPWT are required.

Written informed consent was obtained from the patient for publication and any accompanying images. A copy of the written consent is available for review by the Editor-in-Chief of this journal on request.

## 4 Conclusions

Negative- pressure wound therapy has shown a good result for an infected lower extremity elephantiasis plexiform neurofibroma and as an adjunct as wound dressing for defect closure with STSG.

## Consent

Written informed consent was obtained from the patient for publication and any accompanying images. A copy of the written consent is available for review by the Editor-in-Chief of this journal on request.

## Ethical approval

Ethical approval for this study (Ethical Committee N° NAC 207) was provided by the Ethical Committee of Dr. Hasan Sadikin General Hospital, Bandung, Indonesia on 8 January 2024.

## Funding

This research did not receive any specific funding.

## Author contribution

Lisa Y. Hasibuan: Study concept or design, data collection, data analysis, interpretation, writing the paper

Almahitta Cintami Putri: Study concept or design, data collection, data analysis, interpretation, writing the paper

Graciella Novian Triana Wahjoe Pramono: Study concept or design, data collection, data analysis, interpretation, writing the paper

## Guarantor

Lisa Y. Hasibuan

Almahitta Cintami Putri

Graciella Novian Triana Wahjoe Pramono

## Research registration number

This study does not require registration.

## Conflict of interest statement

The authors declare no conflicts of interest related to this study.
